# Chronic kidney disease progression in glomerular diseases: is hematuria a key risk factor?

**DOI:** 10.1007/s00467-026-07201-0

**Published:** 2026-04-13

**Authors:** Eduardo Gutiérrez, Ángel M. Sevillano, Fernando Caravaca-Fontán, Hernando Trujillo, Jesús Egido, Manuel Praga, Juan Antonio Moreno

**Affiliations:** 1 Department of Nephrology, Instituto de Investigación Hospital Universitario, 12 de Octubre, Madrid, Spain; 2 Instituto de Investigación, Hospital Universitario 12 de Octubre, Madrid, Spain; 3 Renal, Vascular and Diabetes Research Laboratory, IIS-Fundación Jiménez Díaz, Universidad Autónoma Madrid, Madrid, Spain; 4 Spanish Biomedical Research Centre in Diabetes and Associated Metabolic Disorders (CIBERDEM), Madrid, Spain; 5 Department of Medicine, Universidad Complutense de Madrid, Madrid, Spain; 6 Maimonides Biomedical Research Institute of Cordoba (IMIBIC), Hospital Universitario Reina Sofía, Córdoba, Spain; 7 Department of Cell Biology, Physiology and Immunology, University of Cordoba, Campus of International Agri-Food Excellence, ceiA3, 14014 Córdoba, Spain

**Keywords:** Hematuria, IgA nephropathy, Acute kidney injury, Chronic kidney disease, Red blood cells

## Abstract

Hematuria has traditionally been considered a benign sign of glomerular injury, as reported in autosomal dominant type IV collagen-related nephropathy. However, growing clinical and experimental evidence suggests that it may be an active driver of kidney dysfunction. Persistent microscopic hematuria is associated with an increased risk of progression to kidney failure. Likewise, episodes of macroscopic hematuria, particularly in Immunoglobulin A nephropathy, can precipitate acute kidney injury and contribute to chronic impairment of kidney function. Glomerular bleeding may be associated with alterations in the glomerular filtration barrier, allowing erythrocyte leakage into the urinary space. At the molecular level, erythrocyte breakdown products, such as free hemoglobin and heme, induce oxidative stress and inflammation, exerting direct cytotoxic effects on kidney parenchymal cells. In this comprehensive review, we describe the prevalence of hematuria in pediatric patients, elucidate the cellular and molecular mechanisms by which hemoglobin and its heme derivatives promote kidney injury, evaluate the impact of hematuria on long-term kidney outcomes, and discuss emerging therapies against hematuria-induced nephrotoxicity.

**Graphical Abstract:**

**Figure:**
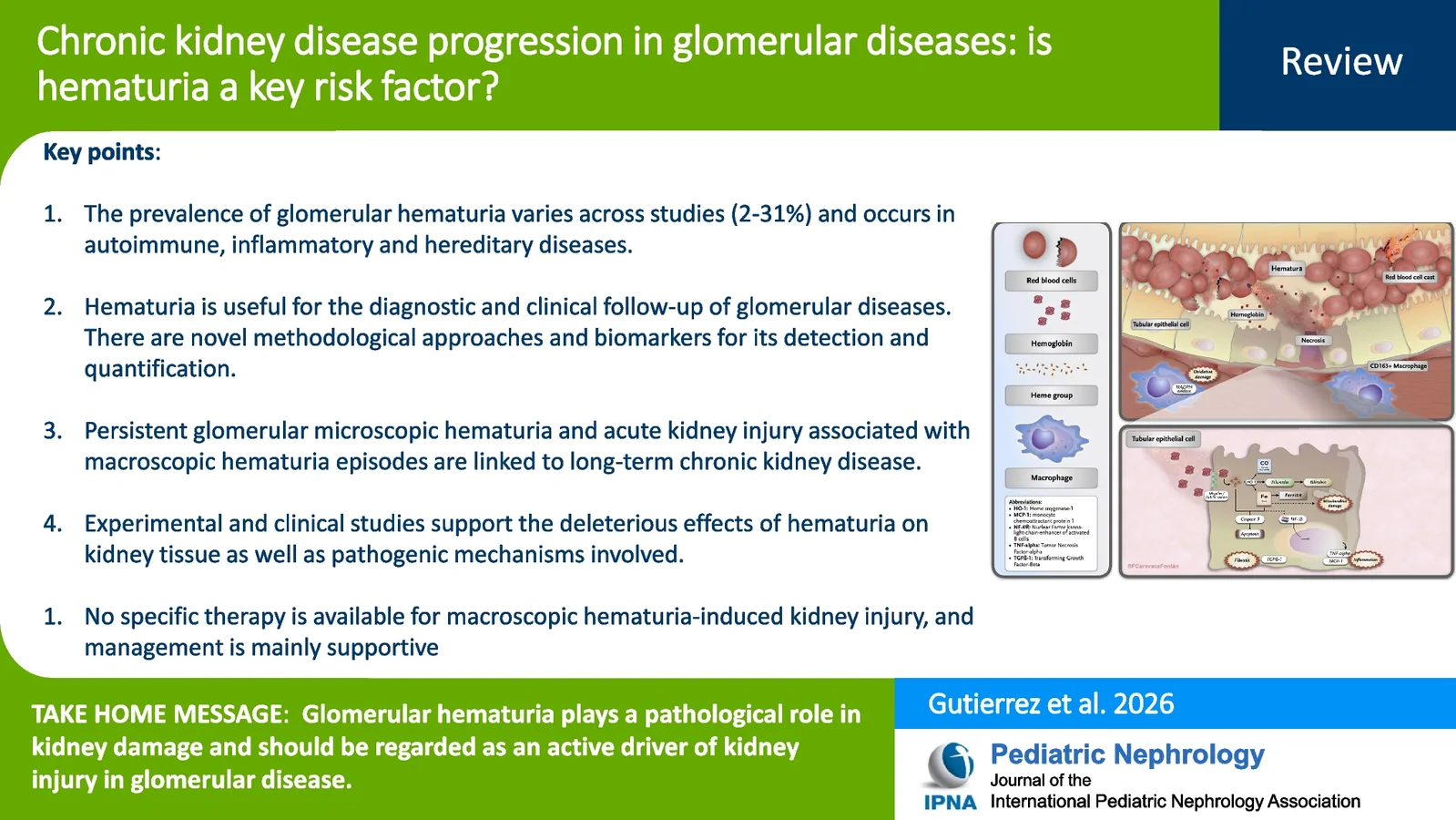
A higher-resolution version of the Graphical abstract is available as  Supplementary information

**Supplementary Information:**

The online version contains supplementary material available at https://doi.org/10.1007/s00467-026-07201-0.

## Introduction

Many kidney and urological disorders, such as urinary tract infections, nephrolithiasis, tumors and glomerulonephritis, can manifest with hematuria. Thus, depending on its origin, hematuria can be classified as glomerular or non-glomerular. In the case of glomerular diseases, hematuria results from disruption of the normal glomerular architecture, allowing the passage of proteins and cellular elements from the capillary lumen into the urine. Consequently, hematuria is a hallmark feature in various glomerular disorders, and its diagnostic significance has been well recognized for decades [1]. However, in some conditions associated with genetic alterations of *COL4A3/A4/A5*, as reported in type-IV collagen-related nephropathy (T-IV CRN), no relation between hematuria and kidney progression was found [2]. Thus, its role as a factor in the progression of glomerular diseases has been scarcely considered, especially when compared to proteinuria/albuminuria. Undoubtedly, technical challenges surrounding a precise and reproducible quantification of hematuria have been a key factor in this oversight [3]. However, recent studies have revitalized the importance of hematuria, not only as a diagnostic marker but also as an indicator of disease activity in various glomerular disorders [e.g., IgA nephropathy (IgAN), antineutrophil cytoplasmic antibody (ANCA)-vasculitis, lupus nephritis and others] [1]. In this way, in the last years a number of studies have demonstrated that hematuria exerts harmful effects on glomerular and tubulointerstitial structures, elucidating the pathogenic pathways responsible for this damage. The aim of this review is to present an updated overview of the methods for quantifying glomerular hematuria, the structural alterations that facilitate its occurrence, its impact on the prognosis of glomerular diseases, and the underlying molecular mechanisms explaining this damage. Finally, it discusses current and emerging treatments aimed at counteracting these effects.

## Definition, detection, and quantification of hematuria

Hematuria is defined by the presence of more than 3 erythrocytes per high-power field [4]. In addition to urological disorders, diseases that affect the renal tubules or interstitium can produce hematuria without primary glomerular injury, leading to isomorphic erythrocytes and absence of casts in the urine sediment [5]. In contrast, glomerular hematuria results from damaged glomerular filtration barrier (GFB) and it is characterized by the presence of acanthocytes in the urine, a type of dysmorphic erythrocytes [6]. In patients with glomerular bleeding, acanthocytes arise as they traverse the GFB. Patients with macroscopic non-glomerular hematuria frequently present red or pink-colored urine often with clots, whereas the urine of patients with macroscopic glomerular hematuria is typically brown [7]. For that reason, it is necessary to do a thorough evaluation of the urinary sediment to determine its origin [3]. The presence of dysmorphic erythrocytes in the urine sediment is the gold standard for discerning the glomerular origin of hematuria. However, manual analysis is a time-consuming process with high interobserver variability. Dipsticks have been routinely used to resolve this limitation; however, they are not able to discriminate against the source of hematuria, provide a semi-quantification of RBCs and can be falsely positive due to RBC hemolysis and many other factors [3]. New automated urinary flow cytometers can quantify small and lysed erythrocytes, detecting glomerular hematuria accurately, although these instruments are not yet widely implemented in routine practice [8]. Adopting these advanced technologies more broadly may enhance determining the origin of hematuria and elucidate its impact on glomerular pathology.

## Prevalence of hematuria and causes of glomerular hematuria

Reported hematuria rates vary widely across studies, depending on the characteristics of the population examined, the detection methods used, and whether a confirmatory test was performed [9]. Microscopic hematuria is common in children and adolescents and is most often detected incidentally during routine urine testing (reported prevalence ranges from 0.5% to 2% in unselected pediatric populations) [10]. Transient microscopic hematuria is frequently observed in children and may be associated with intercurrent illness, exercise, or minor trauma. In contrast, persistent microscopic hematuria (at least two or three positive urine samples over several months) raises greater clinical concern and prompts further evaluation. The diagnostic and prognostic value of microscopic hematuria is strongly influenced by the presence of associated clinical or laboratory findings. Concomitant proteinuria, hypertension, reduced glomerular filtration rate, or abnormal urinary sediment markedly increases the likelihood of an underlying glomerular disorder [11]. Persistent microscopic hematuria in children was associated with a significantly increased relative risk of developing chronic kidney disease (CKD) over long-term follow-up, but the absolute risk remains low. Adolescents and young adults with persistent asymptomatic isolated microscopic hematuria had a hazard ratio of 18.5 for treated CKD compared to those without hematuria, with an incidence of 0.7% over approximately 22 years [12].

**Fig. 1:**
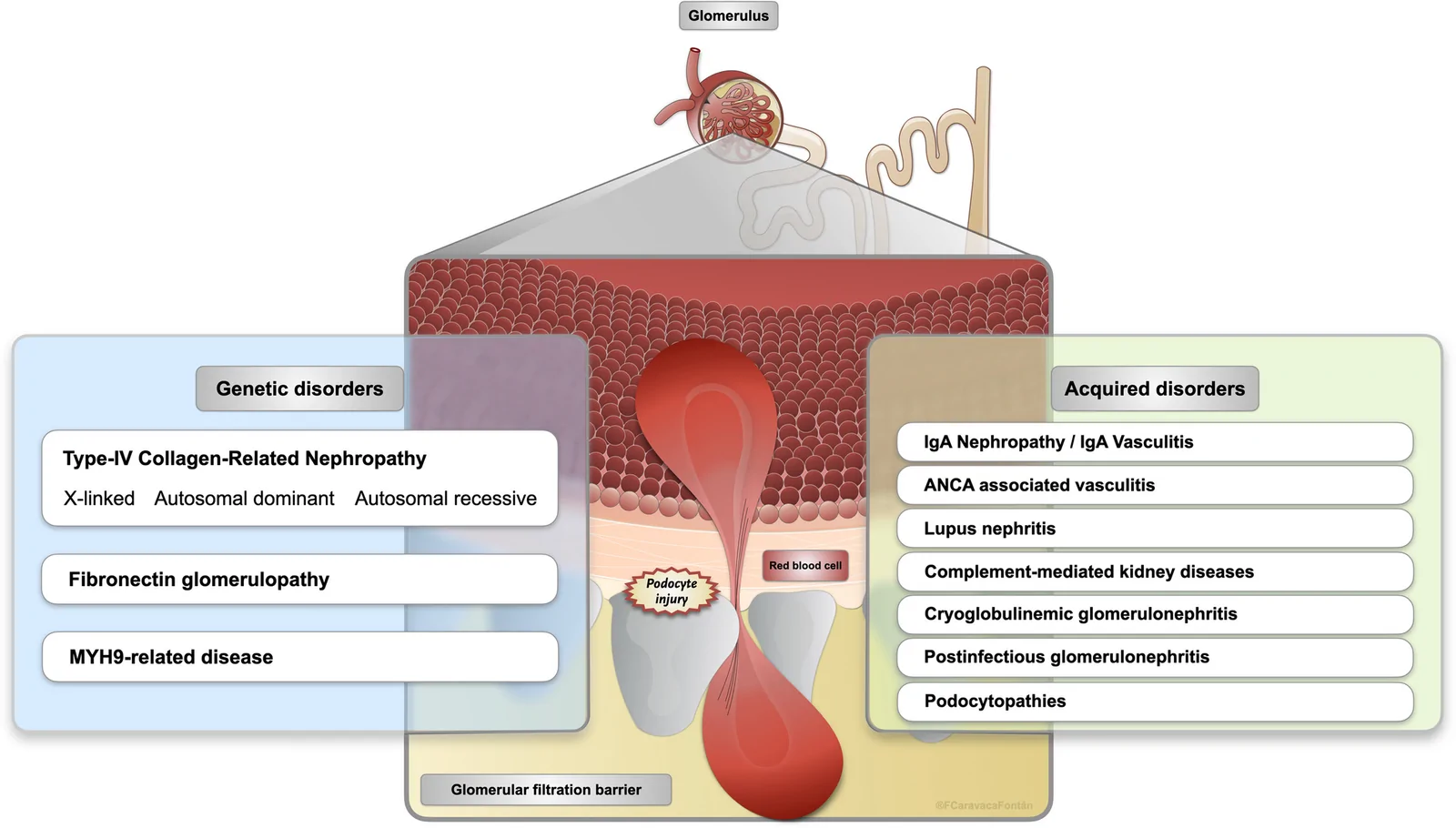
Clinical presentation of hematuria in genetic and acquired kidney disorders

Hematuria is a relatively common finding in patients with CKD, with an estimated prevalence of 15–29% [13]. However, this prevalence varies widely depending on the underlying etiologic cause of CKD. In this way, in specific glomerular diseases such as IgAN or membranoproliferative glomerulonephritis, the prevalence of hematuria is approximately 90% and 80%, respectively [14, 15]. In contrast, hematuria is less commonly observed in diabetic nephropathy and hypertensive nephrosclerosis, where proteinuria tends to be the predominant urinary abnormality [16]. Common causes of glomerular hematuria are shown in Fig. 1. IgAN is the most prevalent cause of glomerular hematuria within primary glomerulopathies, whereas autosomal dominant T-IV CRN is the most frequent genetic cause of glomerular hematuria in the general population [2].

The prevalence of hematuria in kidney biopsy registries is highly variable too. Thus, the prevalence of isolated hematuria and hematuria plus proteinuria ranges from 2.6% to 65% and 4.6% to 46.8%, respectively, as shown in Table 1. These discrepancies may reflect differences in biopsy indication criteria or in the prevalence of kidney diseases in different regions.

**Table 1:** Incidence of isolated hematuria and hematuria + proteinuria in biopsy registries

Country	Isolated hematuria (%)	Hematuria + proteinuria (%)	PMID
Egypt	2.6	–	25394460
Serbia	23.4	15.8	22383346
Italy	19.3	10.8	9509437
China	26.2	4.6	23661591
UK	9.8	–	19729468
Croatia	12.3	–	32102663
China	17	10	18840904
Romania	–	3.3	16249204
China	15.1	24.4	22129070
UK	65	35	9686957
Portugal	28.5	–	17048208
Lebanon	–	46.8	20525974
Spain	4.5	21.7	12198210
France	4.7	19	15327379
Brazil	-	27.2	39928634

## Evidence supporting hematuria as a risk factor for kidney damage

### Microscopic hematuria and kidney disease progression

#### Transplant donors

The presence of microscopic hematuria in a potential kidney donor warrants a systematic and comprehensive evaluation, as it may range from benign etiologies to underlying glomerular diseases that contraindicate donation. The interpretation of this finding in the assessment of donor candidates requires exclusion of urological causes—such as nephrolithiasis, neoplasms, or infections—through appropriate imaging studies and, in individuals over 40 years of age, cystoscopy [17]. When a glomerular origin is suspected, a kidney biopsy is recommended to rule out conditions such as IgAN or T-IV CRN. Recent studies have shown that isolated and persistent microscopic hematuria in donors without pathological findings on biopsy is not associated with post-donation kidney function decline; however, the presence of glomerular disease entails a higher risk of progression. Therefore, microscopic hematuria in a kidney donor represents a finding that justifies exhaustive evaluation and may constitute a criterion for exclusion when significant glomerular pathology is confirmed [18]. Special mention should be made of the study of recurrences of IgAN in donors, reported in 20–60% of IgAN patients, depending on biopsy protocols and duration of follow-up [19]. Living related donors are associated with a higher recurrence risk compared to deceased or unrelated donors [20]. However, mesangial deposits of IgA are frequently found in pretransplant kidney biopsies from donors with no evidence of proteinuria, hematuria, or other manifestations of kidney disease [18]. Reported prevalence of these latent or asymptomatic IgA mesangial deposits varies by population: Asian living donors show 13–29% incidence, while in United States cohorts, rates are 13.2% in living donor kidneys and 24.5% in deceased donor kidneys [21]. In the USA cohort, prevalence differs across ethnicities, being 9.7% among Black donors, 25% among Asian and Hispanic donors, and about 18.2% for Caucasian donors [21]. In Japanese donors, 16% exhibited latent IgA deposition, aligning with autopsy data reflecting general population frequencies (4%–30%). Remarkably, many latent deposits resolve spontaneously: in living donor grafts, 70% of IgA positive grafts become negative within one year post transplantation. Proposed mechanisms include kidney “self-purification” via lymphatics or mesangial proteases [21].

#### Evidence from studies in glomerular diseases

The association and prognostic significance of hematuria has been extensively studied across a wide range of glomerular diseases, including inherited, primary, and secondary forms of glomerulonephritis (Fig. 1).

##### IgA nephropathy and IgA vasculitis

IgAN is the most common primary glomerular disease globally. Hematuria, either microscopic or macroscopic, is a hallmark clinical feature detected in 70–100% of patients at diagnosis [14]. Macroscopic hematuria (MH) often coincides with upper respiratory tract infections [1]. While episodic macroscopic hematuria is not consistently associated with progression, persistent microscopic hematuria – especially when accompanied by proteinuria – is a strong predictor of worse kidney outcomes [22].

Large cohorts from Spain, China, Sweden, and the United States consistently showed that persistent and high-degree microscopic hematuria were independently associated with faster eGFR decline and progression to kidney failure, even after adjusting for confounding factors (Table 2) [14, 23–26]. Conversely, some studies have reported that hematuria remission is associated with improved outcomes. In this way, in a Spanish cohort, patients who achieved hematuria remission (< 5 RBCs/HPF for ≥ 3 years) had significantly slower eGFR decline [14]. Notably, in this study, proteinuria alone without hematuria was associated with a low risk of progression. A subsequent Japanese study involving 74 patients showed that none of those with hematuria remission experienced a 50% eGFR decline, unlike those with persistent hematuria and proteinuria [27]. A recent meta-analysis confirmed an 87% higher risk of kidney failure in those with persistent hematuria [28]. The prognostic significance of the degree of hematuria in IgAN was evaluated by Bobart et al. [29]. In this retrospective study, each incremental increase in median hematuria during follow-up was associated with a significantly faster decline in eGFR, even after adjustment for major clinical and histological confounders (follow-up time, proteinuria, and T score). Hematuria also correlated with histological activity. According to the Oxford classification, it is associated with mesangial hypercellularity (M1), endocapillary hypercellularity (E1), and crescents (C1/C2), with crescents showing the strongest link. However, no association has been found with chronicity markers (S or T scores) [29].

**Table 2:** Clinical studies demonstrating hematuria as an independent risk factor for renal progression in IgA nephropathy

Reference	Study design/analysis	Definition of hematuria	Kidney outcome	Independent association with kidney progression
14	Univariate and multivariate Cox regression	Time-averaged hematuria	Kidney failure	Multivariate HR 2.84 (1.06–7.30); *p* = 0.04
24	Cox proportional hazards regression	Time-averaged hematuria	Doubling of SCr or eGFR < 15 mL/min/1.73 m^2^	Multivariate HR 1.004 (1.001–1.008); *p* = 0.010
25	Time-varying Cox regression	Time-varying hematuria	Kidney failure or ≥ decline 50% decline in eGFR	Multivariate HR 1.63 (1.33–2.01); *p* < 0.001
26	Univariate and multivariate Cox regression	Time-averaged RBC count	Kidney failure or ≥ 50% decline in eGFR	Multivariate OR 2.1 (1.6–2.7); *p* < 0.001
27	Multivariable regression analysis	Presence of hematuria	Kidney function deterioration	OR 2.87; *p* = 0.041

*OR* odds ratio, *HR* hazard ratio, *SCr* serum creatinine

These findings underscore that persistent hematuria reflects ongoing glomerular injury and that its resolution is a meaningful marker of disease control in IgAN.

The presence of pathogenic variants in the *COL4A3/A4/A5* genes in patients with IgAN has been well described in the literature. Indeed, up to 20% of patients initially classified as having familial IgAN were found to carry mutations in genes encoding alpha chains of type IV collagen [30]. Furthermore, some authors propose that the structural alterations in glomerular basement membrane caused by type IV collagen mutations may predispose individuals to mesangial deposition of IgA-IgG immune complexes, potentially contributing to disease pathogenesis in individuals in whom both conditions coexist [31].

IgA vasculitis (IgAV) is the most frequent vasculitis in childhood. Kidney involvement is a major determinant of clinical outcome and typically presents with hematuria and proteinuria. Although isolated hematuria has not been associated with an adverse prognosis in IgAV [32], the coexistence of hematuria and proteinuria, as well as an initial presentation with nephritic syndrome – where hematuria is a defining feature – are well-established predictors of poor kidney outcome [32]. Moreover, hematuria has been associated with the progression of histological lesions in IgAV, being more frequent in advanced pathological stage IV than in early stage I [33]. Taken together, these findings highlight the clinical relevance of hematuria in IgAV.

##### Type-IV collagen-related nephropathy (T-IV CRN)

This syndrome is characterized by microscopic hematuria and, in some cases, progressive kidney failure, hearing loss, and ocular abnormalities. Males with X-linked disease and individuals with autosomal recessive forms typically present with persistent microscopic hematuria. Up to 90% of females with heterozygous X-linked mutations also exhibit hematuria [3]. In autosomal dominant Type-IV CRN, hematuria may be intermittent or persistent and can include macroscopic hematuria episodes, particularly after respiratory infections [34]. Macroscopic hematuria during childhood has been linked to worse kidney outcomes in X-linked T-IV CRN [35].

##### Other familial forms of glomerular hematuria

Fibronectin glomerulopathy, MYH-9 related disorders, and nail-patella syndromes are rare and inherited conditions frequently associated with persistent hematuria, being a key diagnostic clue in the evaluation of these diseases [36].

##### ANCA-associated vasculitis

In ANCA-associated vasculitis, hematuria supports diagnosis and is part of activity scores like the Birminghan Vasculitis Activity score [37]. Persistent hematuria may indicate ongoing disease or other underlying disease, sometimes requiring biopsy repetition [3]. Its predictive value remains uncertain, though dysmorphic RBCs and hematuria may reflect relapse risk [37].

##### Lupus nephritis (LN)

In LN, hematuria and RBC casts have long been used to screen and monitor disease, although biopsy remains the diagnostic gold standard. Isolated hematuria can indicate proliferative LN, but its value in predicting long-term outcomes is limited compared to proteinuria [38].

##### Complement-mediated kidney diseases

C3 glomerulopathy and primary immune complex–mediated membranoproliferative glomerulonephritis are rare complement-driven diseases where hematuria is present in 80–90% of cases [15]. One study linked hematuria intensity to the presence of crescents on biopsy, suggesting a more aggressive phenotype. Persistent hematuria during follow-up was associated with worse kidney outcomes, while remission correlated with a better prognosis [39].

##### Miscellaneous conditions

Hematuria is also a frequent finding in various other glomerular diseases, including monoclonal immunoglobulin-associated glomerulonephritis, cryoglobulinemic glomerulonephritis, anti-glomerular basement membrane disease, and podocytopathies (e.g., minimal change disease, focal segmental glomerulosclerosis, membranous nephropathy). Its presence in these conditions has been significantly associated with poorer kidney outcomes, underscoring its relevance as a marker of disease severity and progression [40].

### Macroscopic hematuria-associated AKI may promote deterioration of kidney function

#### IgAN patients with macroscopic hematuria-associated AKI

Approximately half of patients with IgAN debut with MH and proteinuria following an upper respiratory tract infection, being one of the typical forms of presentation in this condition. This is particularly common in children and adults at the early stage of the disease [41]. Although MH was considered benign in the past, the occurrence of AKI following episodes of macroscopic hematuria is well described, and the prognosis of these episodes may be adverse. In this way, approximately 25% of patients over 50 years old with MH outbreaks and kidney failure at baseline do not recover their baseline kidney function [42]. Although very little is known about its pathogenesis, tubular damage caused by hemoglobin and iron released by RBCs in the tubular lumen is one of the main mechanisms responsible for AKI associated with MH [43]. The duration of MH is a critical factor in the recovery of kidney function, particularly among older patients and those with pre-existing CKD. In fact, elderly patients with CKD are more susceptible to the deleterious effect of these outbreaks. However, the kidney prognosis of pediatric patients with MH-AKI is good. In a recent study, Ishimori et al. reflected that no child with MH-AKI showed deterioration of kidney function at the last study visit [44]. RBC casts are frequently observed in IgAN, and they are a well-recognized indicator of glomerular injury, resulting from the disruption of the glomerular capillary wall and subsequent glomerular bleeding, often accompanied by MH [45]. The presence of RBC casts is usually associated with decreased kidney function and an increased rate of crescents or endocapillary hypercellularity lesions [46]. However, a recent study evaluating the role of RBC casts in IgAN has yielded some contradictory findings. On one hand, patients with RBC casts showed better kidney prognosis; on the other hand, the presence of RBC casts was significantly and independently associated with kidney disease progression [47]. Moreover, the presence of RBC casts was associated with a favorable response to steroid therapy and may potentially serve as a pathological indicator reflecting kidney disease progression. One possible explanation is that RBC casts may be found in early phases of active lesions with minimal chronic damage. Even so, the formation of RBC casts originates from the entrapment of RBCs within the tubular lumen by uromodulin, whose serum and urinary levels constitute a risk factor for severe histopathological injury in the early stage of IgAN [48]. Although most children with IgAN and MH exhibit favorable short-term kidney prognosis, histological analyses reveal significant iron deposits within kidney tubules. This finding may be of clinical relevance, as iron induces chronic kidney injury, as previously discussed.

#### Anticoagulant-related nephropathy

Anticoagulant-related nephropathy (ARN) is a pathological condition characterized by macroscopic glomerular hematuria followed by AKI as a consequence of the use of oral anticoagulants, antiplatelet agents, heparin, and even in pro-hemorrhagic states [49]. Clinical presentation is severe, with up to 40% of patients requiring acute dialysis, and prognosis is poor, with incomplete kidney recovery in 75% of cases, and 30% of patients remain dialysis-dependent. In patients with incomplete recovery of kidney function, 1-year mortality can be close to 40% [50]. ARN is rare in patients without pre-existing or concurrent kidney disease. Indeed, IgAN or IgA-predominant immune complexes in glomeruli are frequent histopathologic findings [51]. It has been suggested that anticoagulants may trigger macroscopic hematuria and AKI, leading to diagnosis in previously unrecognized cases of IgAN [52]. To date, ARN has not yet been reported in the pediatric population. However, given the underlying pathogenic mechanisms, its occurrence cannot be excluded, particularly in those with preexisting glomerular disease or excessive anticoagulation.

### Mechanisms underlying erythrocyte passage through the glomerular filtration barrier

Glomerular hematuria occurs when erythrocytes pass from the capillary lumen through the GFB into urinary space [53]. In healthy conditions, the GFB does not allow the passage of erythrocytes towards the urinary space. However, in glomerular diseases, inflammatory/immune-mediated injury or structural abnormalities of the GFB compromise its sieve function, permitting transient RBC leakage and hematuria [22].

In immune‐mediated glomerular diseases, autoimmune activation or inflammatory mechanisms have been directly linked to the onset of hematuria. Glomerular immune-complexes deposition induces local activation of the complement system and subsequent glomerular injury and hematuria, as observed in IgAN. In a humanized IgAN experimental model expressing human IgA1 and CD89, increased C3 deposition and glomerular inflammation was associated with hematuria [54]. More recently, it was observed that galactose-deficient IgA1 (Gd-IgA1) and specific IgG autoantibodies from patients with IgAN are responsible for complement activation and development of hematuria [55]. In IgAN, immune complex deposition may promote mesangial cell activation and subsequent release of proteolytic enzymes that can degrade glomerular basement membrane (GBM) [56]. Glomerular hematuria is frequently precipitated by respiratory infections, which act as inflammatory triggers that induce overproduction of immune complexes and activate both the alternative and lectin complement pathways in susceptible individuals, as reported in IgAN, ANCA-vasculitis, C3 glomerulonephritis and membranoproliferative glomerulonephritis [15, 57]. ANCA induce neutrophils to degranulate and produce reactive oxygen species (ROS), resulting in endothelial injury [57]. Other immune mechanisms involved in development of hematuria are neutrophil extracellular traps, which promote breakdown of GFB, as reported in experimental anti-GBM autoantibodies-induced glomerulonephritis [58].

Structural defects in the GFB, both congenital or acquired, promote weakness and fragility of this sieving structure leading to hematuria [53]. This mechanism underlies the persistent microscopic hematuria observed in conditions such as T-IV CRN and *MYH9* gene mutations [59]. T-IV CRN is a genetic disorder secondary to pathogenic variants in type IV collagen genes (*COL4A3/A4/A5*). This causes continued expression of fetal *COL4A1* and *COL4A2*, which are more vulnerable to degradation and predispose to develop segmental GBM ruptures [60].

### Pathophysiology of kidney injury associated to hematuria

**Fig. 2:**
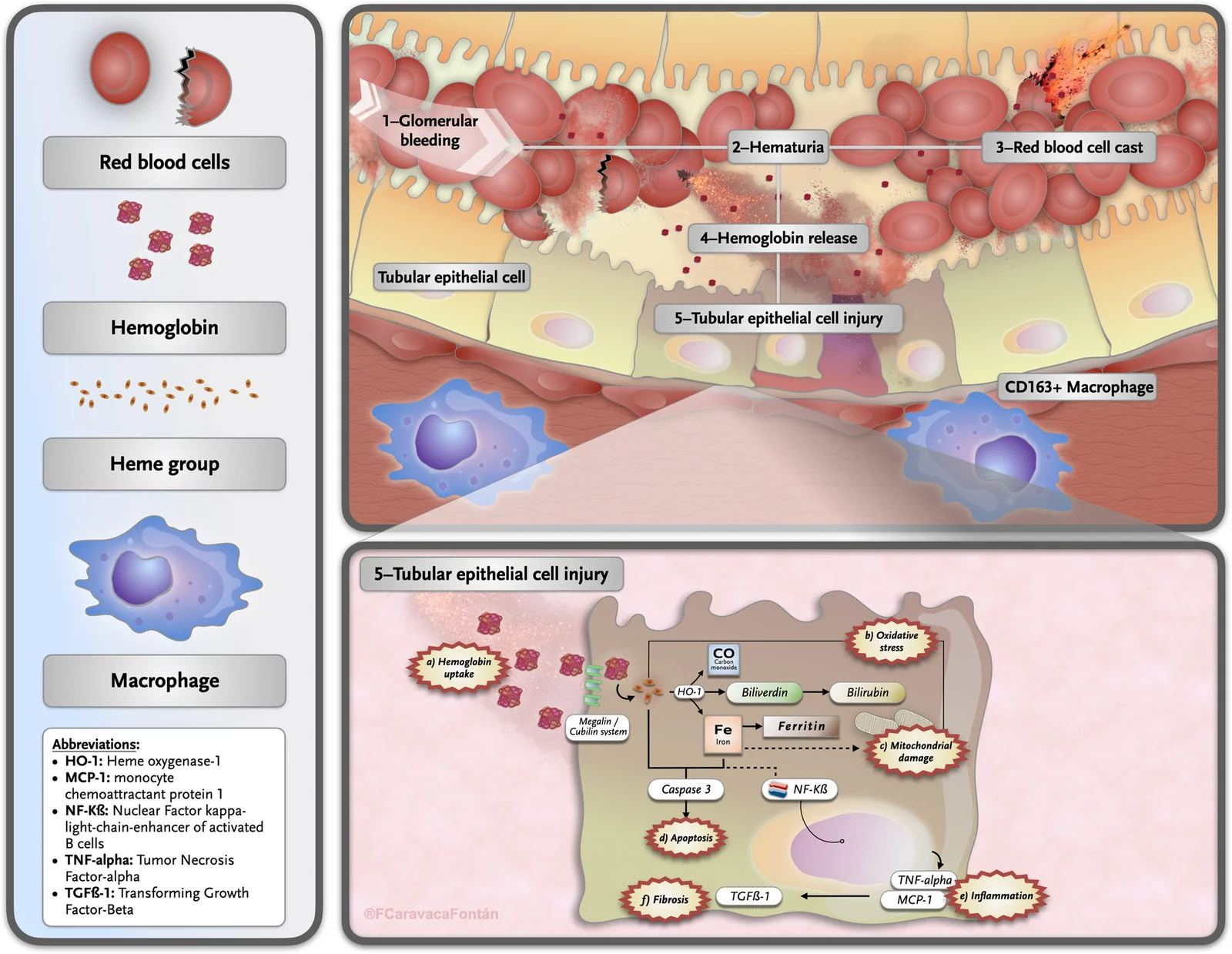
Pathogenic mechanisms involved in hematuria-induced kidney injury. RBC casts obstruct renal tubules, leading to acute tubular necrosis. In addition, hemoglobin released from the intratubular degradation of red blood cells may be uptaken by proximal tubular cells or podocytes (not shown) via the megalin–cubilin receptor complex or degraded within the tubular lumen, leading to the release of heme-containing molecules and eventually free iron. The intracellular accumulation of hemoglobin/heme/iron within kidney cells promotes the generation of reactive oxygen species, mitochondrial injury, caspase activation, and cell death, as well as inflammation and fibrosis. CD163 + macrophages act as scavengers of extracellular hemoglobin and initiate anti-inflammatory and fibrotic pathways to maintain tissue integrity

Hematuria promotes kidney damage through multiple, often overlapping mechanisms (Fig. 2). Studies in kidney biopsies from patients with acute kidney injury associated with macrohematuria (IgAN and ARN) demonstrated the presence of RBC casts obstructing distal and collecting tubules, leading to acute tubular necrosis [42, 49]. In IgAN, obstruction by RBC casts correlated with AKI severity [42]. However, acute tubular necrosis has been observed in patients with macrohematuria in the absence of obstructive casts, suggesting that additional mechanisms contribute to hematuria-induced nephrotoxicity [61]. Because hemosiderosis is frequently observed during macroscopic hematuria [43], it has been proposed that hemoglobin (Hb) and heme released by RBC lysis act as key mediators of kidney damage.

The initial phase of kidney injury is related to direct cytotoxic effects of Hb and heme in kidney cells. Cell-free Hb is uptaken by tubular cells and podocytes through the megalin/cubilin receptor system [62]. Within these cells, Hb dissociates into globin chains and heme, a cytotoxic molecule that directly induces oxidative stress, mitochondrial dysfunction, and cell death [63, 64]. Heme-mediated cytotoxicity is largely driven by iron-dependent oxidative mechanisms. Heme catalyzes Fenton’s reaction, generating hydroxyl radicals that promote lipid peroxidation and drive ferroptosis, an iron-dependent form of regulated cell death implicated in tubular injury [65]. In parallel, heme accumulation disrupts mitochondrial homeostasis, altering mitochondrial ultrastructure and impairing the mitochondrial dynamics, thus exacerbating mitochondrial ROS production [66]. Both tubular cells and podocytes undergo mitochondrial-dependent apoptosis in response to Hb and heme overload, as demonstrated in vivo and in vitro [42, 43, 67].

Endoplasmic reticulum stress further contributes to this direct heme-mediated cytotoxicity, particularly in tubular epithelial cells, as shown by upregulation of unfolded protein response markers, such as activating transcription factor 4 (ATF4), X-Box binding protein 1 (XBP1), and C/EBP homologous protein (CHOP) [66–68].

Beyond these direct toxic effects, heme also initiates secondary inflammatory and fibrotic responses that amplify kidney injury, mainly in tubular epithelial cells. Cell-free heme functions as a damage-associated molecular pattern activating toll-like receptor 4 pathway [69] and NF-κB-mediated expression of proinflammatory cytokines C–C motif ligand 2 (CCL2) and C–C motif ligand 5 (CCL5) and profibrotic mediators, such as connective tissue growth factor (CTGF) and transforming growth factor-beta (TGF-β) [70]. In addition, heme-mediated activation of complement C3/C5 exacerbates kidney inflammation and amplifies kidney injury in heme-overload conditions [71].

To counteract these deleterious effects, tubular cells and podocytes upregulate heme oxygenase-1 (HO-1), which degrades heme into biliverdin, carbon monoxide, and free iron that is subsequently stored by ferritin [72]. Both HO-1 and ferritin are transcriptionally upregulated by factor erythroid 2-related factor 2 (Nrf2), a master regulator of antioxidant responses activated in heme-induced kidney damage [73]. However, when heme accumulation overwhelms these protective systems, extensive tubular and podocyte cell death ensues.

In later stages, surviving tubular cells may adopt a maladaptive pro-fibrotic phenotype, characterized by sustained secretion of CTGF and TGF-β, leading to fibroblast activation and extracellular matrix deposition. In parallel, infiltrating macrophage expressing CD163, the scavenger receptor for extracellular hemoglobin, may further amplify kidney fibrosis, as suggested in patients with AKI and macrohematuria [74].

### Hematuria in clinical practice guidelines

Despite its established association with adverse kidney outcomes, the integration of hematuria into clinical practice guidelines remains limited and heterogeneous. The Kidney Disease: Improving Global Outcomes (KDIGO) 2024 Clinical Practice Guideline for the Evaluation and Management of Chronic Kidney Disease recognized hematuria as a marker of kidney damage and a supportive element in the diagnosis of CKD and its underlying causes. Hematuria is also considered a criterion for referring pediatric patients to a nephrologist to rule out kidney diseases. Furthermore, the same guidelines acknowledge that hematuria may be associated with an increased risk of subsequent CKD development [75]. Similarly, the KDIGO 2021 Clinical Practice Guideline for the Management of Glomerular Diseases recommends regular monitoring of hematuria in various glomerular diseases, particularly in IgAN, due to its prognostic significance, especially when it coexists with proteinuria [76]. However, KDIGO guidelines do not include specific recommendations for screening or follow-up of patients with isolated microscopic hematuria [75].

The National Kidney Foundation’s Kidney Disease Outcomes Quality Initiative (KDOQI), in its commentary on the 2021 KDIGO glomerular disease guideline, highlighted the importance of urine sediment analysis, across all forms of glomerulonephritis. KDOQI also emphasized the potential prognostic value of assessing the magnitude and persistence of hematuria, particularly in diseases such as IgAN and IgA vasculitis [77]. Although it is not the focus of this review, the American Urological Association (AUA) guideline on microscopic hematuria underscored the importance of hematuria as a clinical sign for ruling out urological malignancy, especially in patients over 35 years of age or those with risk factors such as smoking [78]. This highlights hematuria’s role as a potential marker for urinary tract malignancy. In contrast, the American College of Physicians advises against routine screening for hematuria in asymptomatic adults, citing insufficient evidence that such screening improves cancer detection or outcomes [79]. This guideline does not address the potential role of hematuria in the detection or progression of CKD.

### Therapeutic approaches for glomerular hematuria

The treatment of patients with glomerular hematuria depends on the underlying condition responsible for this clinical manifestation [75]. Some authors have proposed using hematuria to guide both the initiation and efficacy of treatment in IgAN [1]. This recommendation is supported by the results of recent clinical trials. For example, in the NEFIGARD trial, treatment initiation with budesonide led to a rapid decrease in microscopic hematuria, which was associated with a significant reduction in proteinuria and a slower decline in kidney function [80]. This pattern of hematuria improvement after the initiation of immunosuppressive therapy has also been described in other studies. However, the specific treatment strategies for IgAN and other glomerulonephritis are beyond the scope of this review.

On the other hand, although it is well established that hemoproteins can induce kidney damage, current clinical guidelines in the management of hematuric glomerulopathies do not recommend therapeutic approaches directed at this pathogenic mechanism. This underscores a substantial translational gap between mechanistic insights obtained from experimental studies and the current lack of clinically applicable therapies targeting heme-mediated toxicity in patients with glomerular hematuria. For example, in patients with IgAN who develop MH-AKI, KDIGO guidelines recommend only supportive care [81]. This is because, to date, no therapy has demonstrated a clear clinical benefit in patients with MH-AKI. Supportive care includes close clinical monitoring, adequate hydration, and urine alkalinization to reduce iron dissociation from the hemoglobin. Beyond the clinical guidelines, in cases of prolonged MH (exceeding 15 days), corticosteroid regimens have been empirically administered in real-world clinical practice; however, these interventions are based on uncontrolled studies, and their outcomes have been inconsistent [48]. Similarly, there is no specific immunosuppression, and results have been inconsistent [81].

In contrast, in other kidney conditions mediated by hemoprotein toxicity, such as rhabdomyolysis or intravascular hemolysis, other therapeutic strategies have been proposed. For instance, the use of iron chelators like deferoxamine or deferiprone has demonstrated beneficial effects in experimental models and clinical practice [82]. Other suggested interventions to prevent hemoprotein-induced kidney injury include the use of hemopexin or acetaminophen [83, 84], as well as Nrf2 inducers [85]. However, these therapeutic approaches have not yet been evaluated in hematuria-related conditions.

In parallel with the current lack of targeted therapies, there is a clear unmet need for biomarkers to determine hematuria-induced kidney injury. Tubular injury markers (e.g., Ngal, Kim-1), heme-handling pathways (CD163), and inflammatory mediators, including complement activation products, may represent promising candidates. Prospective studies integrating hematuria intensity with tubular injury and inflammatory biomarkers may improve disease monitoring and identification of patients at high risk of progression to chronic kidney damage.

## Conclusion

Hematuria primarily represents a consequence of glomerular filtration barrier disruption, reflecting underlying glomerular injury. The pathogenetic mechanisms leading to glomerular hematuria are being elucidated and include immune-mediated damage, complement activation, and structural defects of the GFB. Beyond its established diagnostic value, increasing clinical evidence suggests that hematuria may also play a role in the progression of kidney injury in glomerular diseases. Thus, persistent microscopic hematuria, particularly when accompanied by proteinuria, has been consistently associated with worse kidney outcomes in IgAN, and is widely recognized as a marker of disease activity or relapse in primary and secondary glomerulonephritis, including ANCA-associated vasculitis and LN. Importantly, longitudinal studies indicate that persistent or time-averaged hematuria accelerates decline of kidney function, independently of proteinuria or other confounding factors. Nevertheless, most available data are observational, and the effect of concomitant proteinuria cannot be fully excluded.

Episodes of macroscopic hematuria-associated AKI represent a distinct but clinically relevant scenario, in which glomerular bleeding precedes tubular injury that can lead to incomplete kidney recovery. While the long-term prognosis of such episodes appears favorable in most pediatric cases, irreversible loss of kidney function has been documented, particularly in older patients or those with pre-existing CKD.

Mechanistically, experimental studies provide evidence supporting a direct pathogenic role of hematuria downstream from the initial glomerular injury. Once erythrocytes enter the tubular lumen, hemoglobin and heme can exert direct cytotoxic effects on parenchymal renal cells, triggering oxidative stress, mitochondrial dysfunction, inflammation, and fibrosis. Therefore, hematuria may be both a marker of glomerular injury and disease activity, as well as a pathogenic mediator of kidney injury.

Despite these advances, critical knowledge gaps remain. Studies are needed to standardize the quantification and reproducibility of glomerular hematuria, better define its prognostic influence, and search for therapies that can counteract its deleterious effects on the progression of glomerular diseases.

## Supplementary Information

Below is the link to the electronic supplementary material.

Graphical abstract (PPTX 6.47 MB)
